# Development of Cultured Muscles with Tendon Structures for Modular Bio-Actuators

**DOI:** 10.3390/mi12040379

**Published:** 2021-04-01

**Authors:** Takuto Nomura, Masaru Takeuchi, Eunhye Kim, Qiang Huang, Yasuhisa Hasegawa, Toshio Fukuda

**Affiliations:** 1 Department of Micro-Nano Mechanical Science and Engineering, Nagoya University, Nagoya 4648603, Japan; takeuchi@mein.nagoya-u.ac.jp (M.T.); yasuhisa.hasegawa@mae.nagoya-u.ac.jp (Y.H.); 2 Department of Mechatronics Engineering, Meijo University, Nagoya 4688502, Japan; kim@meijo-u.ac.jp (E.K.); tofukuda@meijo-u.ac.jp (T.F.); 3 Intelligent Robotics Institute, School of Mechatronical Engineering, Beijing Institute of Technology, Beijing 100081, China; qhuang@bit.edu.cn

**Keywords:** bio-robot, bio-actuator, cultured muscle

## Abstract

In this article, we propose a new actuator named the modular bio-actuator (MBA). The MBA has two tendon structures made of polydimethylsiloxane (PDMS) at both ends of the bio-actuator. The MBA can be easily handled and fixed on an artificial micro-robot body to increase its design flexibility and output power. The tendon structures were connected to a bio-actuator in the form of a chain structure, and the connection between the tendon structures and the bio-actuator was maintained for more than three weeks. The contraction length of the MBA was linearly increased when the DC voltage applied to the MBA was increased. The MBA contracted over 200 µm when a DC voltage of 10 V and 1 Hz was applied to the bio-actuator. The output power of the MBA was measured using a PDMS cantilever, and the total output power of the MBA increased linearly when multiple MBAs were stacked on a PDMS cantilever. This study was aimed at improving the design flexibility and controllability of micro-robots and bionic systems.

## 1. Introduction

### 1.1. Conventional Bio-Robots

Bio-robots are hybrid systems that integrate biological components with artificial components [1]. Bio-robots are expected to be the next generation of robots, having the flexibility of living organisms, high energy efficiency through direct conversion of chemical energy into kinetic energy, self-repair, high controllability, and high accuracy. Bio-robots are expected to be pertinent to the development of soft robotics and nano-scale mechatronics.

Bio-actuators have been actively applied to actuate bio-robots recently [2,3,4,5,6,7,8,9,10,11,12,13,14,15]. A bio-actuator is an actuator that uses biological muscles. An artificial body and bio-actuators can be integrated into a bio-robot. Many of the bio-robots developed so far used thin-film shapes to culture and actuate muscle cells on the film surface. Park et al. developed a soft-robotic ray [2] using rat heart muscle cells. Kim et al. developed sheet-shaped muscle using C2C12 skeletal muscle cells [3]. The muscle cells were cultured on the thin film in these studies, and they were fixed automatically on the surface of a bio-robot. Such bio-robots using thin film could quickly achieve fixation and actuation of muscle cells. However, the bio-robots’ design, output force, and lifetime were limited, mainly because the bio-actuators could not be replaced once the bio-robots were fabricated.

The bio-actuators on a thin film have limitations: when the bio-actuators are fabricated on a thin film, they gradually contract and finally lose the ability to contract after a few days [16]. The design of bio-robots is limited when the bio-actuators are placed on a thin film. The maximum thickness of the bio-actuator is limited to a few hundred micrometers to prevent necrosis [17], but the area of bio-actuators should be significant to increase the contraction force. To overcome these limitations, we propose a modularized bio-actuator. Modularized bio-actuators can be tightly fixed when they are not actuated. The design flexibility of bio-robots can be increased if the bio-actuators are placed at an arbitrary position by the modularization. The contraction force can be increased by stacking multiple modularized bio-actuators.

In this study, rigid anchors (tendon structures) were added to both ends of a bio-actuator to maintain fixation during the incubation period and repeatedly fix the bio-actuator on a micro-robot. We named the modularized cultured muscle with tendon structures a modular bio-actuator (MBA).

Several studies have been conducted on the construction of three-dimensional (3D) bio-actuators with tendon structures so far. For example, Morimoto et al. developed a cell sheet using a collagen gel as a scaffold [16]. They produced a bio-actuator by laminating and fixing cell sheets on pillars during cell culture. The bio-actuators were combined with linkage mechanisms to construct bio-robots. Therefore, the bio-actuators could not be detached or re-attached from the body of the bio-robot. Yamasaki et al. developed bio-actuators using collagen gel as a scaffold [18]. They applied an acellular tissue to the cultured muscle as a tendon structure. The acellular tissue was made by removing elastin fibers from the aorta obtained from dissecting pigs. Those bio-actuators were modularized but dissection of a pig is required for the preparation of each bio-actuator. Akiyama et al. added an anchor to bio-actuators using silicone sponge and nonwoven nylon mesh [19]. The nonwoven nylon mesh anchors fixed bio-actuators for more than three weeks. The silicone sponge anchor fixed the bio-actuators for about two to four days, and the bio-actuator ruptured at the junction. Hence, these bio-actuators were modularized. However, the bio-actuators were attached only on the anchor surface by the surface force and the fixation force largely depended on the materials of the fixation place.

The purpose of our research was to make exchangeable bio-actuators. In this paper, we present the MBA by describing the combination of 3D bio-actuators with artificial tendon structures. Mouse skeletal muscle C2C12 cells were used for preparing the bio-actuators, and we cultured C2C12 cells with the tendon structures on a culture template. An extracellular matrix (ECM) was used for the 3D culture of C2C12 cells in the culture template.

### 1.2. Concept of the MBA

**Figure 1:**
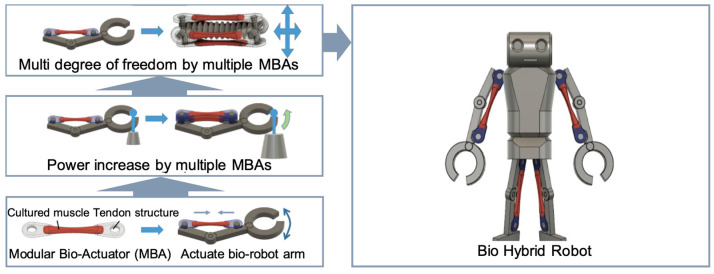
Concept of the modularized bio-actuator (MBA) and its applications for micro-robots.

Figure 1 shows the concept of the MBA. The MBA has two tendon structures and it can be fixed on the body of a micro-robot. A micro-robot force can be increased by stacking the multiple MBAs, and the degree of freedom of a micro-robot can be increased by assembling multiple MBAs on a micro-robot. Hence, the MBA can increase design flexibility and power control compared with conventional bio-actuators. The MBA can be applied to develop micro-robots with multiple degrees of freedom (DoFs) and contribute to the development of cyborg and bionic systems in the future.

**Figure 2:**
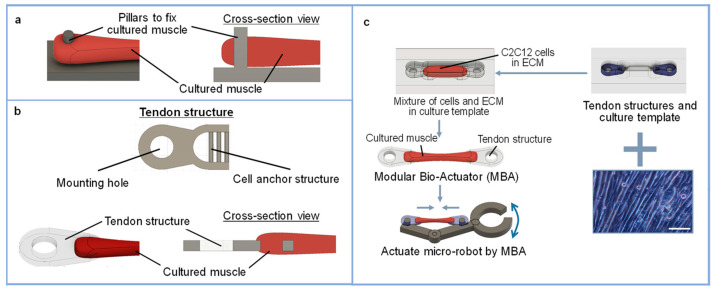
Overview of the MBA: (**a**) fabrication procedure of the MBA (scale bar: 100 μm); (**b**) design of the tendon structure proposed in this study; (**c**) conventional fixation method for cultured muscle using pillars.

The modular bio-actuator system proposed in this study is a modular system that uses tendon structures and culture templates (Figure 2). Generally, pillars have been used to fix and stretch bio-actuators [20,21] because a bio-actuator’s fixation could be easily achieved when pillars are used. However, bio-actuators can be easily detached from a pillar when a force is applied in the pillar axis direction (Figure 2a). In our study, the tendon structures were attached to both ends of the bio-actuator to overcome such limitations. The MBA can be repeatedly fixed on a micro-robot and removed from the micro-robot using the tendon structure. The tendon structure was designed to have two holes: one hole can be used to connect with the bio-actuator (named the mounting hole) and the other hole can be used to fix the bio-actuator on a robot (named the cell anchor structure) (Figure 2b). They were also designed to be anchored to the culture template to maintain the bio-actuator’s entire length during the incubation period. In this study, the extracellular matrix Matrigel (Corning Life Science, New York, NY, USA) was used as a scaffold for the 3D culture of myocytes. Matrigel was used on a culture template to fabricate arbitrarily shaped bio-actuators (Figure 2c).

## 2. Materials and Method

### 2.1. Materials of MBA

In this study, we used C2C12 cells to fabricate MBAs. A multi-nucleation medium was used to make bio-actuators. The growth medium was prepared with Dulbecco’s Modified Eagle’s Medium (DMEM) with 100 units/mL penicillin G, 100 μg/mL streptomycin sulfate, and 10% fetal bovine serum (FBS). The DMEM with 100 units/mL penicillin G, 100 μg/mL streptomycin sulfate, and 2% horse serum (HS) was used as the multi-nucleation medium. As a scaffold for the 3D culture of cells, a Matrigel (Corning) basement membrane matrix made from a mouse-derived ECM was used. Polydimethylsiloxane (PDMS, DuPont Toray Specialty Materials) was used as the material for the culturing template and tendon structures due to its high bio-compatibility and easy processing and molding. The molds of culture templates and tendon structures were 3D printed with Acrylonitrile- Butadiene- Styrene (ABS) resin.

### 2.2. Preparation of Culture Template and Tendon Structures

It is known that cultured skeletal muscle improves elasticity and tension during cell culture. Adequate drag force is necessary for skeletal muscle growth during cell culture. If the skeletal muscle is cultured without drag force, bio-actuators begin to contract excessively due to their tension and cannot function with contractility. Therefore, the muscle must be strongly fixed to a mechanical stretcher for the application of adequate drag force during cell culture. Generally, pillars have been used to fix and stretch bio-actuators [21,22]. Fixation of a bio-actuator can be easily achieved when pillars are used. However, the bio-actuator can be easily detached from the pillar when a force is applied in the pillar axis direction (Figure 2c).

In this study, we developed a new tendon structure. The tendon structure had two parts, a mounting hole and a cell anchor structure, as shown in Figure 2b. The mounting hole was used to fix the pillar of the culture template, another MBA, or other robot parts. The muscle cells were cultured and fixed by the cell anchor structure, as shown in Figure 2b. The cell anchor structure was designed to be a comb-like structure. This structure can increase cell adhesion and the detachment of the bio-actuator from the tendon structure can be prevented. Since the bio-actuator grows to engage the cell anchor structure, it can be expected to show the same anchoring force for the same shape, even if the material is changed. The tendon structures were tightly fixed on the culture template by placing the tendon structures in the holes at both ends of the culture template. The drag force can be applied to muscle cells during cell culture by the fixation of tendon structures.

**Figure 3:**
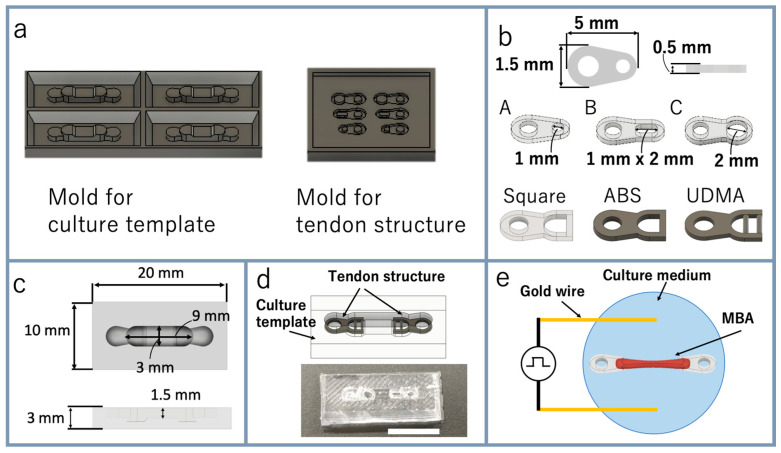
Fabrication of tendon structures and a culture template: (**a**) 3D print prototype of culture template and tendon structures as molds of polydimethylsiloxane (PDMS); (**b**) design of the tendon structure; (**c**) design of culture template; (**d**) combination of tendon structures and culture template (upper image: 3D CAD image; lower photo: fabricated culture template with tendon structures) (scale bar: 10 mm); (**e**) experimental setup for applying voltage to the MBA using gold wire.

Bio-actuators consisting only of myocytes are too soft and vulnerable to handle them manually. The MBA can be easily handled manually by picking up the tendon structures. The mold prototypes of culture templates and tendon structures were made using 3D CAD software (Figure 3a). The base material and PDMS curing agent were mixed at 10:1 and poured into the molds of the tendon structures and the culture template. The PDMS was degassed using a vacuum defoamer and desiccator. It was cured in an oven at 70 °C for about 5 h, and the cured PDMS was taken from the molds. For the sterilization, the culture template and the tendon structures were immersed in 70% ethanol for 1 h, then irradiated with ultraviolet (UV) light them for 1 h. After that, 2-methacryloyloxyethyl phosphorylcholine (MPC) polymer Lipidure-CM 5206 (NOF America Corporation, White Plains, NY, USA) was coated on the surface of the PDMS culture template to prevent adhesion of cells. The tendon structure had a hole for the cell anchor structure, and the cells mixed in the ECM were placed into the hole to fix the bio-actuator on the tendon structures. The tendon structures were designed with a thickness of 0.5 mm (Figure 3b). Six different tendon structures were designed for different shapes of the cell anchor structure. A, B, C, and Square were the four tendon structures with different sizes and shapes for the cell anchor structure’s hole. All four tendon structures were prepared with PDMS. The square tendon structures were additionally made of ABS resin and urethane dimethacrylate (UDMA). The length of the MBA was 9 mm in this culture template (Figure 3c), and this was the most extended length used in this study. The tendon structures were tightly fixed in the culture template, as shown in Figure 3d.

### 2.3. Preparation of Myoblast Cells

C2C12 cells were seeded in a culture dish filled with growth medium and cultured in a 5% CO_2_ incubator at 37 °C. The passage was carried out when the cells covered 80% of the bottom of a culture dish. The culture medium in the culture dish was removed, and the bottom surface of the culture dish was washed with 10% phosphate buffer solution (PBS). After that, cells were detached from the culture dish using 0.25% trypsin EDTA. After detaching cells, the growth medium was mixed and pipetted well. The detached C2C12 cells were gathered by centrifugation, and the passaging was completed by seeding gathered cells on a new culture dish.

### 2.4. Fabrication of MBA

The C2C12 cells were used for fabricating MBA when they covered more than 90% of the culture dish bottom. The culture medium in the culture dish was removed, and the dish bottom was washed with 10% PBS. C2C12 cells were detached from the culture dish with 0.25% trypsin EDTA. The growth medium was added to the culture dish and pipetted well. After that, solitary C2C12 cells were gathered by centrifugation. The centrifuge tube was cooled with 70% ethanol at 2 °C for 5 min. The Matrigel and the C2C12 cells were mixed to a cell concentration of 1.5 × 10^8^ cells/mL. Then, 10 μL of Matrigel containing the C2C12 cells was placed on the culture template. The tendon structure was fitted on the culture template, and another10 μL of Matrigel containing the C2C12 cells was added to the culture template. The cells were cultured in the 5% CO_2_ incubator at 37 °C for 10 min to promote the Matrigel polymerization. After complete polymerization of the Matrigel, the growth medium was added and the cells were incubated for three days in the incubator. Then, the growth medium was exchanged with the multi-nucleation medium, and the multi-nucleation of the C2C12 cells was promoted by replacing the multi-nucleation medium every two days.

### 2.5. Actuation of Bio-Actuator

Electric pulse stimulation was applied to the bio-actuator when the bio-actuator showed tensile force from differentiation induction. A function generator was used to apply electric pulse stimulation. A square wave of 10 V, 2% duty ratio, and 1 Hz frequency was used as the electric pulse. The electric pulses were applied by placing conductive wires into the medium. Gold wires with a diameter of 0.5 mm were used as conductive wires due to their chemical stability (Figure 3e).

## 3. Results

### 3.1. Fabrication of MBA

**Figure 4:**
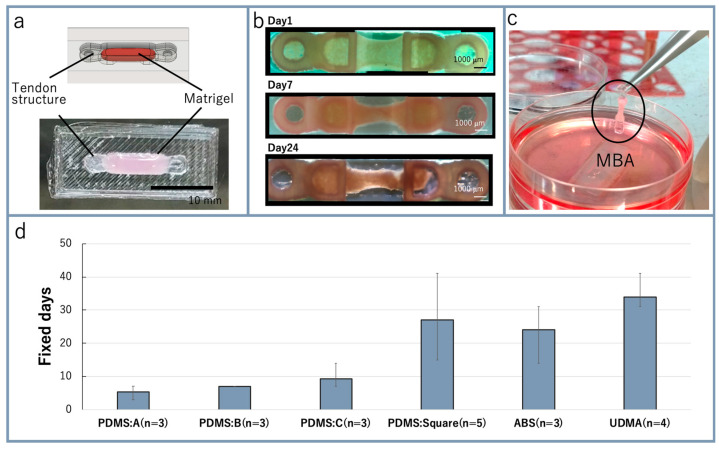
Results for the fabricated MBA: (**a**) Matrigel containing C2C12 cells put into the culture template (scale bar: 10 mm); (**b**) bio-actuators on days 1, 7 and 24 after the start of culture; (**c**) handling of MBA by tweezers; (**d**) number of days of bio-actuator fixation possible for each tendon structure.

The Matrigel poured into the culture template was polymerized in the incubator (Figure 4a). Figure 4b shows photo images of the bio-actuator from 1 day to 24 days after the starting of cell culture. The tendon structure was handled with tweezers when the MBA was picked up and placed on a new culture template (Figure 4c). During the culturing, a tensile force was generated from the bio-actuator fixed on the tendon structures. The bio-actuator was stretched continuously and adequately with the tensile force generated from it. After three days, the bio-actuator growth and the connection between the tendon structure and bio-actuators became stable through the cell anchor structure. Four days after the starting of cell culture, the growth medium was exchanged with the multi-nucleation medium to promote the multi-nuclearization of C2C12 cells in Matrigel. The muscles cultured for two weeks were picked from the culture template and placed on a new culture template. The developed MBA showed enough strength to fix onto and detach from the culture template repeatedly. To acquire the experimental results, these fixation and detachment repetitions were undertaken about five times.

The tendon structures were prototyped with materials such as PDMS, ABS, and UDMA to extend the number of days that the bio-actuator could maintain fixation. The results for the making of the MBA with each tendon structure are shown in Figure 4d. All of the tendon structures based on squares were able to maintain fixation of the bio-actuator over two weeks. UDMA showed the highest performance, and it was used for subsequent experiments.

In our method, the bio-actuator was mechanically fixed by the tendon structure. Our tendon structure could fix bio-actuators without using cell adhesion, and the tendon structure with a square shape for the cell anchor structure fixed the bio-actuator for over three weeks. Compared with conventional studies, such as those that involved fixation of bio-actuators for two to four days on a silicone sponge anchor and more than three weeks on a nonwoven nylon mesh anchor [18], our method achieved almost the same stable fixation of bio-actuators without depending on the materials of the anchor.

### 3.2. Electrical Stimulation to Bio-Actuator

**Figure 5:**
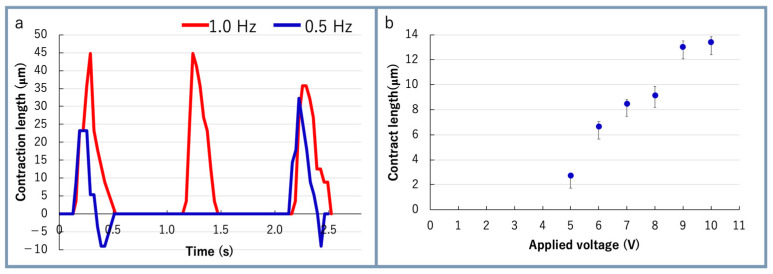
Experimental results of MBA actuation: (**a**) contraction length of bio-actuator when electrical stimulation at different frequencies was applied to the MBA; (**b**) relationship between the applied voltage and contraction length of bio-actuator (*n* = 6).

The electrical stimulation was applied to the bio-actuators after one week. For electrical stimulation, a square wave pulse was used with 10 V, 2% duty ratio, and 1 Hz frequency. The bio-actuator repeatedly contracted at about 1 Hz when the electric pulse was applied to the bio-actuator. When the pulse frequency was changed to 0.5 Hz, the bio-actuator repeatedly contracted at about 0.5 Hz. The maximum local contraction of the bio-actuators was 45 μm compared with natural length (Figure 5a). The result indicated that the differentiation of C2C12 cells was promoted and they were partially multi-nucleated by culturing cells in Matrigel with tendon structures. When the applied voltage was increased stepwise from 5 to 10 V, the MBA’s contraction force was also increased according to the voltage (Figure 5b).

**Figure 6:**
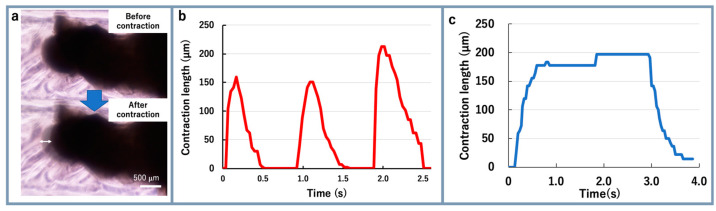
Experimental results of MBA actuation: (**a**) before and after contraction of a bio-actuator, when one end of the bio-actuator was cut off from the tendon structure; (**b**) contraction length of a bio-actuator when one end of the bio-actuator was cut off from the tendon structure; (**c**) contraction length of the bio-actuator when electrical stimulation with a 20 Hz pulse was applied.

A twitch is a short contraction of a cultured muscle and can follow an input stimulation frequency under about 5 Hz. A tetanus, obtained by superimposing the twitches, is a prolonged contraction generated by an input stimulation frequency around about 10 Hz [16,23]. In the next step, one end of the bio-actuator was cut off from the tendon structure (Figure 6a) to measure the MBA twitch and tetanus contractions. When the electro-stimulation was applied to the MBA, the MBA contracted over 200 µm (Figure 6b). The contraction was maintained with 20 Hz stimulation (Figure 6c). Hence, the MBAs showed the same characteristics as conventional skeletal muscles [16,23].

For the advancement of the multi-nucleation of bio-actuators, it is clear that longer time cultivation is necessary. Moreover, to improve the contraction rate of bio-actuators, the orientation of muscle fibers should be aligned in one direction. With our method, the bio-actuator could be cultured for three weeks by fixing it on the tendon structures. The orientation of muscle fibers could be aligned in one direction through the contraction force generated from cells. Hence, the MBA can advance the multi-nucleation of bio-actuators.

The contraction length of a muscle is proportional to its total length, and the contraction force is proportional to its cross-sectional area. Therefore, it is possible to improve the contraction force by connecting the MBAs in parallel. It is also possible to improve the contraction force by connecting the MBAs in series. However, the contraction rate of the bio-actuator used for the MBA was 5% of the total. In contrast, it is about 20% of the muscle in living animals. The preparation method for MBAs needs to be improved to increase their contraction rate. MBAs use Matrigel as a scaffold for a steric culture for the muscle cells. The volume of Matrigel occupies more than half of the bio-actuator. Although Matrigel is essential as a scaffold, it acts as a resistance to the bio-actuator’s contraction. Therefore, it is necessary to increase cell density in the bio-actuator by reducing the amount of Matrigel.

### 3.3. Stacking Multiple MBAs

**Figure 7:**
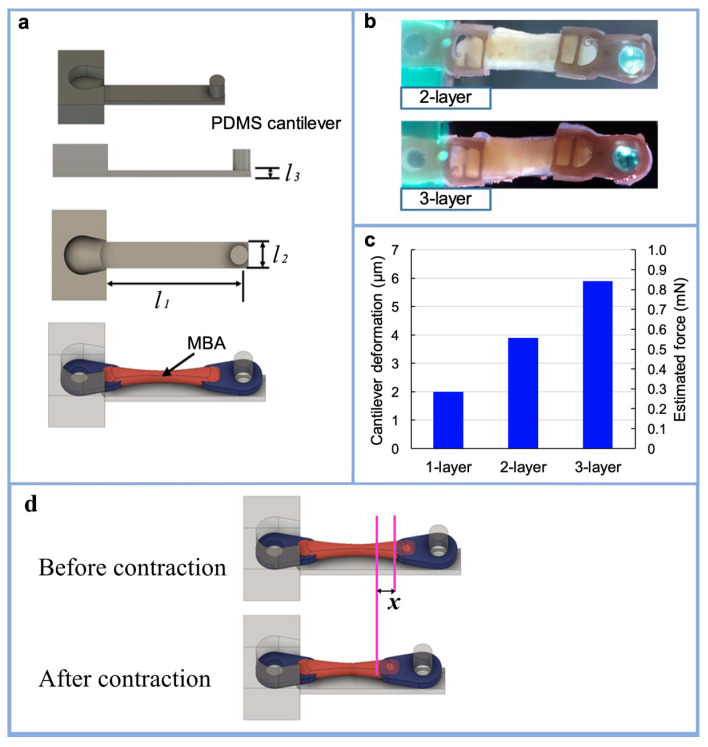
Stacking of multiple MBAs on the PDMS cantilever: (**a**) design of PDMS cantilever to estimate the force generated from MBAs; (**b**) stacked MBAs on the PDMS cantilever; (**c**) experimental results of PDMS cantilever deformation from stacked MBAs and estimated force generated from the stacked MBAs with different numbers of layers; (**d**) calculation of the contraction force for the MBA.

As shown in Figure 7d, the PDMS cantilever was deformed by the MBA’s contraction connected to the arm. The MBA’s contraction force can be estimated by measuring the cantilever deformation and the PDMS Young’s modulus. The dimensions of the cantilever were 12 mm × 2 mm × 0.5 mm. Multiple MBAs were stacked, layer by layer, on one PDMS cantilever (Figure 7b), and electrical stimulation was applied to measure the PDMS cantilever deformation. The applied voltage was set at 10 V in the experiments. Up to three MBAs were stacked layer by layer on a single PDMS cantilever, and the deformation of the cantilever was observed under a microscope for the different numbers of stacked MBAs. The contraction force of each MBA was calculated from the tendon structure edge movement *x* (Figure 7d). Assuming a Young’s modulus of 1.8 MPa for PDMS with a base material-to-hardener ratio of 10:1, the contract force of the MBA was derived with the following formula.

(1) $$F_{k}=\frac{P\times x_{k}\times l_{1}}{l_{1}\times l_{2}\times l_{3}}$$

In where *F_k_* is the contraction force of the MBA; *x_k_* is the amount of deformation of the cantilever or the amount of tendon structure movement; *k* is the number of MBAs stacked on the cantilever; *l*_1_, *l*_2_, and *l*_3_ are the length, width, and thickness of the cantilever, respectively (Figure 7a); and *P* is the Young’s modulus of the PDMS. The values of *x*_1_, *x*_2_, and *x*_3_ in the experiment were as follows: $x_{1}=2.0\;\mu m,\;x_{2}=3.9\;\mu m,x_{3}=5.9\;\mu m$. From the above, the contraction force was estimated for *F*_1_, *F*_2_, and *F*_3_ as about 0.30 mN, 0.59 mN, and 0.88 mN, respectively. Figure 7c shows the PDMS cantilever deformation for each number of layers of MBAs. The results indicate that the force generated from the MBAs was almost proportional to the number of stacked MBAs.

In the MBA contraction, the applied voltage was just a trigger of the contraction and was not an energy source for contraction [24]. The electrical stimulation was applied to the MBA with the same voltage and duration regardless of the number of stacked MBAs. Nevertheless, stacked MBAs showed different contraction forces with different stacking numbers. Therefore, the cultured muscle got energy for contraction from the culture medium.

## 4. Conclusions

We fabricated bio-actuators with tendon structures and named them modularized bio-actuators. The square tendon structure made of PDMS showed the bio-actuator’s stable connection over more than three weeks, and the tendon structure made of UDMA could be kept connected to the bio-actuator for over 30 days. These MBAs can be fixed on the skeletons of artificial micro-robot structures using the tendon structures. The MBAs were handled and fixed on different places in our experiments. Thus, the proposed MBA has high modularity as a new bio-actuator. This high modularity means that bio-robots can be designed in any shape and from any material arbitrarily.

Multiple MBAs were connected layer by layer to one PDMS cantilever, and electrical stimulation was applied to measure the PDMS cantilever deformation. Different numbers of stacked MBAs provided force proportionally. Thus, we showed that a parallel connection of the MBAs could increase the force of bio-actuator contraction. This study was intended to contribute to the development of new bio-robots, cyborg systems, and bionic systems actuated by bio-actuators.
